# Transperineal Ultrasound Combined with Contrast-Enhanced Ultrasound Enema for Diagnosis and Surgical Planning in Rectovaginal Fistula: A Prospective Study

**DOI:** 10.3390/diagnostics16142209

**Published:** 2026-07-15

**Authors:** Jia Huang, Yao Zhang, Huan Pu, Bin Sun, Xingyue Huang, Jun Zhang, Qing Deng, Qing Zhou

**Affiliations:** Department of Ultrasound Imaging, Renmin Hospital of Wuhan University, Wuhan 430060, China; huangyujia1010@163.com (J.H.);

**Keywords:** rectovaginal fistula, transperineal pelvic floor ultrasound, ultrasound contrast agent enema, pelvic floor injury, diagnostic imaging, surgical planning

## Abstract

**Background/Objectives**: Rectovaginal fistula (RVF) is a challenging condition that can substantially impair quality of life and often requires individualized surgical management. Accurate preoperative imaging is essential for identifying fistulous tracts, assessing pelvic floor involvement, and guiding surgical planning. However, conventional imaging modalities, including magnetic resonance imaging (MRI), may still have limitations in detecting small, occult, intermittently patent, or anatomically complex fistulas, particularly under static imaging conditions. This study aimed to evaluate the diagnostic performance of transperineal pelvic floor ultrasound (TPUS) combined with ultrasound contrast agent enema (UCAE) and its potential value in preoperative assessment of RVF. **Methods**: In this prospective single-center study, 62 women with surgically confirmed RVF were enrolled between January 2022 and April 2025. All patients underwent TPUS combined with UCAE before surgery, while subsets also underwent contrast-enhanced CT, contrast-enhanced MRI, or barium enema according to clinical indications. Imaging findings were compared with intraoperative findings as the reference standard. Fistula detection, morphological classification, size, anatomical location, pelvic floor injury assessment, and concordance between ultrasound-based surgical proposals and actual surgical procedures were systematically analyzed. Comparative analyses with conventional imaging modalities were performed based on available non-paired subgroups. **Results**: UCAE detected RVF in 60 of 62 patients, yielding a detection rate of 96.8%. In the available comparative cohort, UCAE demonstrated higher detection rates than conventional imaging modalities, particularly in small and complex fistulas. UCAE showed high agreement with intraoperative findings in terms of fistula morphology, size, and anatomical location. TPUS provided additional and complementary information regarding levator ani and anal sphincter injuries and yielded higher sensitivity and accuracy in pelvic floor injury assessment within the studied cohort. Ultrasound-based surgical proposals showed directional concordance with final intraoperative decisions in 88.7% of cases. No adverse events were observed during UCAE. **Conclusions**: TPUS combined with UCAE appears to be a safe and feasible preoperative imaging approach for RVF. By integrating fistula detection, anatomical classification, pelvic floor assessment, and surgical planning, this combined ultrasound approach may serve as a complementary imaging strategy to conventional imaging modalities, particularly for small or intermittently patent fistulas. Further multicenter studies and standardized imaging protocols are warranted to validate its clinical utility and generalizability.

## 1. Introduction

Rectovaginal fistula (RVF) is an abnormal communication between the rectum and vagina, resulting in the passage of gas, feces, or purulent discharge through the vaginal canal. This condition can substantially impair quality of life and is frequently associated with recurrent infection, psychological distress, and social dysfunction [1,2]. RVF may arise from various etiologies, including obstetric injury, inflammatory bowel disease, pelvic surgery, anorectal procedures, and oncologic treatment [1]. Although surgical repair remains the mainstay of treatment, recurrence is not uncommon [3]. Therefore, accurate preoperative assessment of fistula anatomy is critical for selecting an appropriate surgical strategy and reducing the risk of incomplete repair.

Conventional imaging modalities, including endorectal or endovaginal ultrasound, computed tomography (CT), magnetic resonance imaging (MRI), and barium enema, are commonly used for RVF assessment. Among these, MRI is generally regarded as the reference imaging modality for complex anorectal and rectovaginal fistulas due to its superior soft-tissue contrast and multiplanar capability. However, in clinical practice, these imaging methods may still have limitations in detecting small, occult, intermittently patent, or complex fistulas, particularly when the rectal and vaginal walls are closed or semi-closed under resting conditions [4,5,6]. In addition, comprehensive preoperative evaluation should ideally provide not only fistula detection, but also detailed information regarding fistula morphology, anatomical location, pelvic floor involvement, and surgical accessibility.

Transperineal pelvic floor ultrasound (TPUS) combined with ultrasound contrast agent enema (UCAE) may provide a complementary imaging approach for preoperative RVF evaluation. TPUS enables real-time and dynamic assessment of the rectum, vagina, anal sphincter complex, and levator ani muscle [7], while UCAE enhances acoustic contrast through retrograde administration of ultrasound contrast agents, thereby improving the visualization of fistula patency under pressure-dependent conditions [8]. This pressure-sensitive imaging mechanism is particularly useful for detecting intermittently patent or functionally concealed fistulous tracts that may not be apparent under resting conditions. Together, TPUS and UCAE may integrate anatomical visualization, functional patency assessment, and preoperative surgical mapping.

In this prospective study, we enrolled 62 patients with surgically confirmed RVF who underwent preoperative TPUS combined with UCAE. Using intraoperative findings as the reference standard, we evaluated the diagnostic performance of this combined ultrasound approach and its value in guiding surgical planning for RVF, with particular attention to its role as a complementary tool within a multimodal imaging strategy.

## 2. Materials and Methods

### 2.1. Study Design and Patients

This prospective single-center study enrolled 62 consecutive female patients with surgically confirmed rectovaginal fistula (RVF) between January 2022 and April 2025 at our institution. The inclusion criteria were as follows: (1) clinical suspicion of RVF based on typical symptoms, including passage of gas, stool, or purulent discharge through the vagina; (2) preoperative evaluation with transperineal pelvic floor ultrasound (TPUS) combined with ultrasound contrast agent enema (UCAE); (3) subsequent surgical confirmation of RVF with complete operative records; and (4) availability of complete clinical and imaging data. The exclusion criteria were as follows: (1) concurrent severe pelvic disease or malignancy; (2) inability to tolerate the ultrasound examination; (3) known allergy to ultrasound contrast agent; and (4) absence of surgical treatment during the study period. This study was designed as a prospective real-world diagnostic accuracy study. All imaging examinations were performed as part of routine clinical workflow, and only patients who completed both imaging assessment and surgical treatment were included in the final analysis. The study was approved by the Ethics Committee of Renmin Hospital of Wuhan University (approval No. WDRY2025-K211). Written informed consent was obtained from all participants.

### 2.2. Instruments and Imaging Methods

Ultrasound examinations for TPUS and UCAE were performed using a GE LOGIQ E20 system (GE Healthcare, Chicago, IL, USA) equipped with a C1-6VN convex array probe (1–6 MHz) and an L3–12 linear probe (3–11 MHz).The ultrasound contrast agent SonoVue (Bracco, Milano, Italy) was prepared by diluting 5 mL of the agent with normal saline to form a microbubble suspension, which was further mixed with 150 mL of normal saline. Bowel preparation was performed 30 min before the ultrasound examination using a rapid enema. Contrast-enhanced CT was performed using a Siemens SOMATOM Force dual-source scanner (Siemens Healthineers, Forchheim, Germany), and contrast-enhanced MRI was performed using a GE SIGNA Architect 3.0 T scanner (GE Healthcare, USA). Digital barium enema was performed using a Shimadzu SONIALVISION G4 fluoroscopy unit (Shimadzu, Kyoto, Japan), when clinically indicated. All imaging examinations were performed according to routine clinical indications using standard institutional protocols.

#### 2.2.1. TPUS Examination

**Figure 1:**
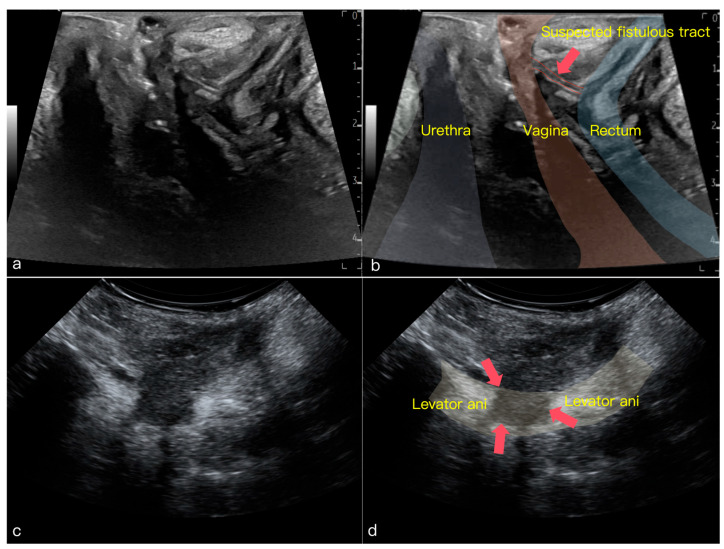
Typical findings of transperineal pelvic floor ultrasound in rectovaginal fistula (RVF). (**a**) Mid-sagittal TPUS image showing the anatomical relationship between urethra, vagina, and rectum (unannotated). (**b**) The same mid-sagittal image with annotations; the red arrow indicates a suspicious fistula tract between the vagina and rectum. (**c**) Parasagittal TPUS image depicting the morphology and continuity of the levator ani muscle (unannotated). (**d**) The same parasagittal image with annotations; the red arrow indicates a discontinuity in the hypoechoic muscle ring, consistent with levator ani injury.

Patients were examined in the supine lithotomy position. In the midsagittal plane, the rectum, anal canal, vagina, and surrounding structures were systematically evaluated for the presence of suspicious fistulous tracts. The probe was then tilted laterally to obtain parasagittal planes for visualization of the bilateral levator ani muscles. Dynamic assessment of levator ani integrity and contractile function was performed during voluntary pelvic floor contraction. The probe was subsequently rotated 90° counterclockwise into the coronal plane and tilted posteroinferiorly to obtain transverse views of the anal canal. During voluntary anal sphincter contraction, cranial and caudal sweeping of the probe was performed to assess the integrity of both the internal and external anal sphincters (Figure 1).

#### 2.2.2. UCAE Examination

**Figure 2:**
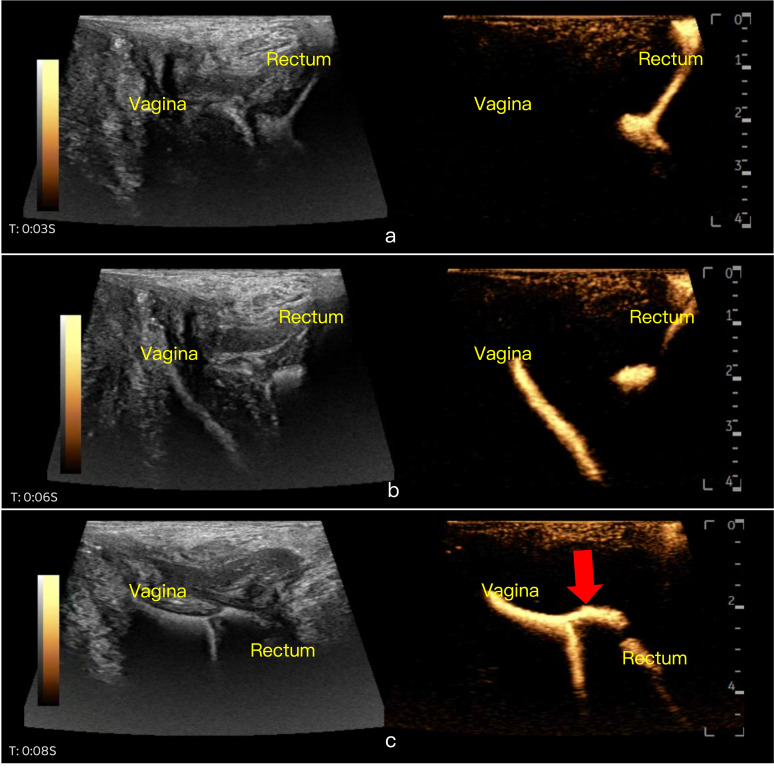
Real-time dynamic observation during contrast-enhanced ultrasound enema. (**a**) At T: 0:03 s, mid-sagittal TPUS image showing contrast delineating the rectal lumen. (**b**) At T: 0:06 s, contrast agent entering the vaginal cavity. (**c**) At T: 0:08 s, after probe angle adjustment, thecontrast agent delineates the course of the rectovaginal fistula (indicated by the red arrow).

**Figure 3:**
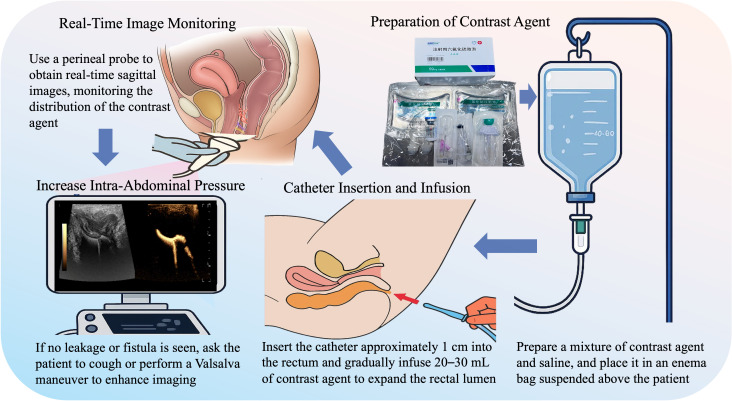
Schematic diagram of the contrast-enhanced ultrasound enema procedure. This diagram outlines the enema ultrasound contrast imaging procedure. After preparing the contrast agent and catheter insertion, the contrast is infused to expand the rectal lumen. Real-time monitoring is conducted with a perineal probe, capturing sagittal pelvic floor images in dual-image mode to observe the contrast distribution within the rectum. The focus is on detecting contrast leakage into the vagina and assessing fistula location, course, and patency. If no leakage is seen, intra-abdominal pressure is increased for enhanced imaging. In the catheter insertion panel, the red arrow indicates the insertion of the enema catheter through the anus into the rectum.

Patients were placed in the left lateral decubitus position. The prepared contrast–saline mixture was placed in an enema bag suspended 40–60 cm above the patient’s hips. A catheter was inserted approximately 1 cm into the rectum, and the contrast–saline mixture was infused in phases at approximately 10 mL/s. Initially, 20–30 mL was instilled to distend the rectal lumen, followed by slow infusion of the remaining 100–150 mL. During infusion, the transperineal probe was used to obtain real-time sagittal images. With a contrast-specific dual-image display, the distribution of the contrast agent was monitored to identify leakage into the vagina, delineate the fistula course, and evaluate fistula patency (Figure 2). If no fistulous communication was observed, patients were instructed to cough or perform the Valsalva maneuver to increase intra-abdominal pressure. All examinations were dynamically recorded for subsequent analysis (Figure 3).

#### 2.2.3. Additional Imaging (CT, MRI, and Barium Enema)

Contrast-enhanced CT was performed using a standard abdominal protocol. MRI was performed using T1-weighted imaging (T1WI), T2-weighted imaging (T2WI), and contrast-enhanced sequences in accordance with routine clinical indications. Digital barium enema was performed with slow infusion of 200–300 mL of diluted barium sulfate suspension. These examinations were performed in a subset of patients based on clinical indications and availability, reflecting real-world clinical practice rather than a predefined comparative imaging protocol. All imaging findings were assessed for fistula detection, anatomical location, tract morphology, and involvement of adjacent pelvic structures using a unified evaluation framework.

### 2.3. Image Analysis and Diagnostic Criteria

All imaging examinations were independently reviewed by two experienced radiologists/sonographers with expertise in pelvic floor imaging. Disagreements were resolved by consensus. All evaluators were blinded to surgical findings. For CT and MRI, RVF was diagnosed based on the presence of a direct abnormal communication between the rectum and vagina or imaging findings suggestive of fistula formation, such as gas, fluid tracks, or continuity between adjacent luminal structures, with or without contrast enhancement. For UCAE, a positive diagnosis was defined as visualization of microbubble contrast passing from the rectal lumen into the vagina or perineal region through a fistulous tract. Pelvic floor injury was evaluated using both MRI and TPUS. On MRI, injury was defined as discontinuity, thinning, or scarring of the levator ani muscle or anal sphincter complex. On TPUS, pelvic floor injury was defined as interruption of the hyperechoic muscular ring, discontinuity of muscle fibers, or absence of contraction during dynamic assessment.

### 2.4. Classification Criteria

**Figure 4:**
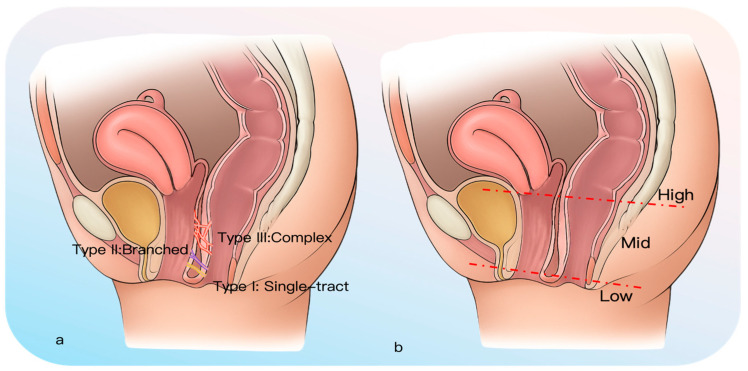
Schematic diagram of rectovaginal fistula (RVF) classification. (**a**) Morphological classification showing Type I (single tract), Type II (branching tract), and Type III (multiple or complex tracts), with Types II and III defined as complex fistulas. (**b**) Anatomical classification depicting fistula openings on the rectal and vaginal sides at high, middle, or low levels.

Fistulas were classified based on imaging findings with respect to morphology, size, and anatomical location. Morphological classification included three types: Type I (single tract), Type II (branching tract), and Type III (multiple or complex tracts) (Figure 4a) [9]. Fistula size was categorized as small (<0.5 cm), medium (0.5–2.5 cm), or large (>2.5 cm), based on the maximum diameter measured on imaging [10].Anatomical location was classified as high, middle, or low according to the level of the rectal opening, with the vaginal opening used as an auxiliary anatomical reference when necessary (Figure 4b) [11,12,13,14].

### 2.5. Preoperative Combined Ultrasound Evaluation and Surgical Planning

All patients underwent combined TPUS and UCAE prior to surgery. Based on ultrasound findings, fistula morphology, size, anatomical location, and pelvic floor structures were systematically evaluated. Two experienced colorectal surgeons independently reviewed the ultrasound results and proposed preliminary surgical recommendations. In cases of disagreement, consensus was reached through discussion. Surgical recommendations were made in accordance with published guidelines [15,16,17,18] and institutional experience (Table 1), which represents an institutional synthesis of typical decision-making patterns rather than a validated algorithm. It should be emphasized that preoperative ultrasound findings were used as an adjunct to clinical decision-making rather than the sole determinant of surgical strategy. Final surgical procedures were determined intraoperatively based on direct surgical findings and comprehensive clinical assessment, including fistula etiology, tissue quality, prior surgical history, inflammatory status, Crohn’s disease, radiation history, and surgeon experience. To explore the supportive role of preoperative ultrasound, agreement between imaging-based surgical suggestions and actual surgical procedures was evaluated. In this context, concordance was defined as whether ultrasound findings provided an anatomically consistent reference to the final surgical approach, rather than exact procedural matching. This analysis was exploratory and aimed to assess the supportive role of ultrasound in surgical planning.

### 2.6. Intraoperative Reference Standard

Intraoperative findings were used as the reference standard for the confirmation of RVF, as well as for the assessment of fistula morphology, size, anatomical location, and pelvic floor involvement.

### 2.7. Outcome Measures

The primary outcome measures included: (1) detection rate of fistulas; (2) concordance of fistula morphology, size, and anatomical location with intraoperative findings; (3) detection of complex fistulas; (4) accuracy of fistula orifice localization; (5) accuracy of pelvic floor injury assessment; (6) concordance between preoperative ultrasound-based surgical proposals and actual surgical procedures; and (7) safety of the examination.

### 2.8. Statistical Analysis

Statistical analyses were performed using SPSS software (version 26.0; IBM Corp., Armonk, NY, USA). Continuous variables were expressed as mean ± standard deviation (SD), and categorical variables were presented as counts and percentages. Comparisons of detection rates were performed using the chi-square test or Fisher’s exact test, as appropriate. Agreement between ultrasound-based assessments and intraoperative findings was evaluated using concordance analysis. Receiver operating characteristic (ROC) curve analysis was used to evaluate the diagnostic performance of imaging modalities for pelvic floor injury assessment. ROC analyses were performed only for imaging modalities with sufficient data available for pelvic floor injury assessment. Sensitivity, specificity, accuracy, and area under the curve (AUC) were calculated. A two-sided *p*-value < 0.05 was considered statistically significant. Intraoperative findings served as the reference standard for all diagnostic performance analyses. Comparisons between imaging modalities were performed using available clinical subgroups according to routine imaging allocation and were interpreted in an exploratory context.

## 3. Results

### 3.1. Baseline Characteristics

A total of 62 female patients with surgically confirmed RVF were included in this study. The patients were 25–68 years old, with a mean age of 41.5 ± 9.7 years, and the mean BMI was 27.0 ± 8.4 kg/m^2^. The etiologies of RVF included obstetric injury in 35 patients (56.5%), inflammatory bowel disease in 8 patients (12.9%), tumor-related therapy in 10 patients (16.1%), and anorectal surgical complications in 9 patients (14.5%). Disease duration was <20 months in 38 patients (61.3%) and ≥20 months in 24 patients (38.7%). Regarding previous management, 14 patients had received conservative treatment, 18 had undergone prior surgical repair, and 6 had received other therapies, including biologic therapy (Table 2). Additional imaging examinations were performed in subsets of patients based on clinical indications, including CT in 24 patients, MRI in 26 patients, and barium enema in 6 patients.

### 3.2. Fistula Detection

UCAE achieved a fistula detection rate of 96.8%. In the available clinical subgroups, UCAE showed higher detection rates than contrast-enhanced CT, MRI, and barium enema. Exploratory pairwise comparisons indicated statistically significant differences between UCAE and each conventional imaging modality (all *p* < 0.05). No statistically significant differences were observed among CT, MRI, and barium enema in detection performance. (Table 3).

### 3.3. Diagnostic Performance of MRI and TPUS in Pelvic Floor Injury Assessment

In the available subgroup for pelvic floor injury assessment, TPUS yielded higher sensitivity, accuracy, and AUC values than MRI for detecting both levator ani and anal sphincter injuries. The numerical differences were more pronounced for levator ani injury than for anal sphincter injury. MRI yielded lower sensitivity and AUC values for levator ani injury than TPUS within the evaluated cohort. (Table 4).

### 3.4. Value of Preoperative Ultrasound in Guiding Surgical Planning

Combined TPUS and UCAE provided detailed preoperative information on fistula morphology, anatomical location, and pelvic floor involvement, which was used as an adjunct in preoperative clinical assessment. Based on ultrasound findings, preliminary surgical recommendations were made for all patients. Final surgical decisions, however, were determined by comprehensive clinical evaluation, including fistula etiology, tissue condition, inflammatory status, prior surgical history, and surgeon judgment. Preoperative ultrasound-based proposals showed agreement with final surgical procedures in 88.7% of patients (55/62). Discrepancies were observed primarily in cases with severe fibrosis, complex fistula tracts, or extensive scarring. No adverse reactions or procedure-related complications were observed during UCAE, and all examinations were well tolerated.

### 3.5. Accuracy of Preoperative Imaging in Morphological Classification and Complex Fistula Identification

**Figure 5:**
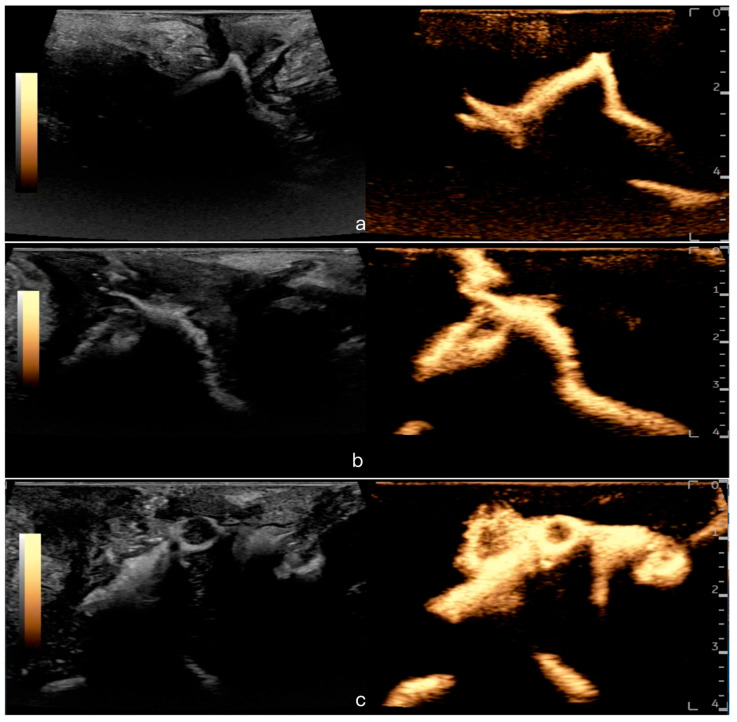
Ultrasound contrast agent enema (UCAE) features of different morphological types of rectovaginal fistula (RVF). (**a**) Type I (single tract) showing a solitary, continuous fistula tract. (**b**) Type II (branching tract) depicting a primary tract with branching secondary tracts. (**c**) Type III (multiple or complex tracts) illustrating multiple intertwined tracts.

UCAE demonstrated good concordance with intraoperative findings in the assessment of fistula morphology, size, and anatomical localization (Figure 5). In the available comparative cohort, UCAE findings more closely approximated intraoperative distributions than CT, MRI, and barium enema, particularly for small tracts and type II/III complex fistulas. Overall differences among imaging modalities were observed in the concordance of preoperative classification with intraoperative findings (χ^2^ = 23.91, df = 3, *p* < 0.001). The anatomical classification based on UCAE findings was generally consistent with intraoperative observations (Table 5).

## 4. Discussion

Rectovaginal fistula (RVF) is a complex condition that requires cross-disciplinary management, involving both colorectal surgery and gynecology. It is characterized by an abnormal tract between the rectum and vagina, often resulting from obstetric trauma, inflammatory bowel disease, pelvic surgical complications, or oncologic therapy [1]. The deep pelvic location, complex anatomy, and frequent presentation as narrow, occult, or branching tracts make accurate preoperative imaging important for precise localization and surgical planning [19,20]. Incomplete assessment increases the risk of intraoperative omissions and recurrence, with reported healing rates ranging from 20% to 100%, and recurrence rates as high as 90% [21,22]. Therefore, reliable preoperative evaluation is generally considered important for reducing recurrence and improving long-term outcomes.

Conventional imaging modalities, including endorectal ultrasound (ERUS), contrast-enhanced CT, MRI, and barium enema, continue to be widely used in clinical practice. ERUS is convenient but may be limited by probe coverage, particularly in high or complex fistulas [5]. CT can demonstrate contrast extravasation but is generally less sensitive for small or intermittently patent tracts. Moreover, radiation exposure limits its use in reproductive-age women. MRI provides superior soft-tissue contrast and is valuable for delineating fistula relationships with pelvic floor musculature, scarring, and abscesses, although its sensitivity for very small tracts may be limited in selected cases [4]. Barium enema allows fluoroscopic visualization of leakage but is affected by overlapping structures and variable filling pressure, which can reduce detection of subtle fistulas.

In this cohort, UCAE demonstrated a high fistula detection rate (96.8%) and good concordance with intraoperative findings in terms of fistula morphology, size, and anatomical localization. In the available exploratory subgroup analyses, UCAE yielded higher detection values than CT, MRI, and barium enema. Among 51 intraoperatively confirmed small fistulas, UCAE identified 49, compared with 6 and 10 identified by CT and MRI, respectively. For complex type II and III fistulas, UCAE depicted fistula tract course and patency in most cases, whereas CT and MRI identified fewer such tracts in this cohort. These differences may be related to the use of intrarectal microbubble contrast under controlled pressure combined with real-time imaging, which may facilitate visualization of fistula orifices and tract courses. Compared with water- or gel-based enemas [23,24], microbubble-enhanced enema may improve luminal contrast and reduce interference from stool or gas. Additionally, maneuvers such as coughing or Valsalva may increase rectovaginal pressure gradients and improve detection of subtle tracts. The technique was safe and well tolerated, with no adverse reactions observed [25].

**Figure 6:**
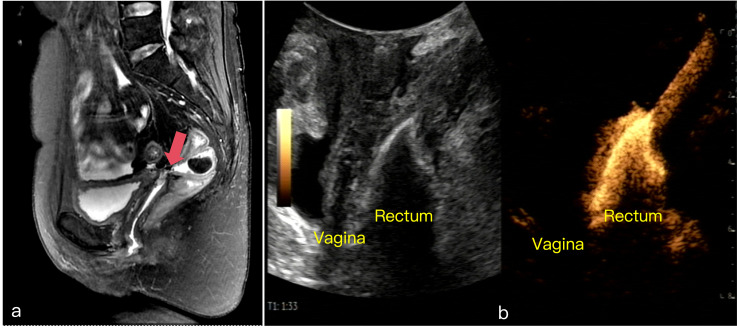
A case of rectovaginal fistula at the vaginal stump: MRI detection and UCAE miss. (**a**) Contrast-enhanced MRI in the sagittal plane clearly delineates a fistulous tract at the vaginal stump (arrow). (**b**) UCAE image showing no fistulous tract, likely due to its high anatomical location.

However, UCAE missed two high- and mid-level small fistulas in this cohort, both of which were detected by MRI (Figure 6). This finding highlights the complementary role of MRI, particularly for high or atypical fistulas. While UCAE may be effective for detecting superficial, occult, or dynamically patent tracts, MRI provides superior soft-tissue contrast and a broader field of view, which is particularly useful for evaluating fistula morphology in relation to the anal sphincter complex, as well as associated fibrosis or collections relevant to surgical planning. Overall, these findings support a complementary rather than competing role of UCAE and MRI in RVF evaluation.

Beyond fistula detection, RVF often coexists with sphincter complex injury or pelvic floor dysfunction, which may contribute to anal incontinence [26,27]. In cases with sphincter defects, concurrent sphincteroplasty may improve structural support and functional outcomes [16,17,18]. Therefore, successful surgical repair requires not only accurate identification of the fistulous tract but also comprehensive evaluation of pelvic floor anatomy. In this study, combined TPUS and UCAE provided complementary information on both fistula anatomy and pelvic muscle structures. TPUS yielded higher sensitivity and accuracy values than MRI within the available cohort for assessing levator ani and anal sphincter integrity. These findings suggest that TPUS may be a useful modality for evaluating pelvic floor injury and assisting in preoperative surgical planning, including adjunctive sphincteroplasty or reconstruction.

Surgical decision-making for RVF remains complex, as tract morphology, size, location, and pelvic floor involvement all contribute to the selection of an appropriate surgical approach. In this study, we summarized an ultrasound-informed surgical planning framework based on combined ultrasound assessment, which showed relatively high concordance (88.7%) between preoperative proposals and intraoperative decisions. Compared with previous studies focusing mainly on isolated imaging features [4,5,24,25], this framework illustrates how ultrasound findings may provide structured anatomical information to support preoperative planning.

UCAE, combined with TPUS, may provide a dynamic, radiation-free, and well-tolerated imaging approach for RVF evaluation. While ultrasound-based techniques have been explored in anorectal disease research [28,29], this study suggests their potential applicability in RVF for detecting and classifying fistulas. UCAE demonstrated relatively high sensitivity and accuracy in this cohort. Its accessibility, low cost, and relatively short learning curve [30] may support its use in selected clinical settings, particularly where MRI availability is limited or in patients for whom MRI is less feasible. In addition, combined ultrasound may also be useful for postoperative follow-up and assessment of recurrent disease, contributing to an integrated preoperative and postoperative imaging strategy.

This study has several limitations. First, it was a single-center prospective cohort study with a relatively limited sample size, which may introduce selection bias and limit the generalizability of the findings. Second, postoperative follow-up was not included, and therefore associations between imaging findings and long-term outcomes such as recurrence or functional recovery could not be evaluated. Third, MRI examinations were performed as part of routine clinical practice, and imaging parameters were not fully standardized across all patients, which may have influenced comparative performance. Therefore, comparisons among imaging modalities should be interpreted cautiously as observational subgroup analyses rather than definitive head-to-head comparisons. Fourth, in some complex cases with extensive fibrosis or altered anatomy due to prior surgical interventions, discrepancies were observed between preoperative imaging and intraoperative findings, highlighting the challenges of imaging evaluation in advanced or recurrent disease.

In summary, the present findings suggest that combined TPUS and UCAE may provide practical preoperative information on fistula patency, morphology, anatomical level, and pelvic floor involvement in patients with RVF. Rather than serving as a standalone replacement for existing imaging modalities, this combined ultrasound approach may be incorporated into a multimodal assessment framework to support individualized surgical planning. Further studies are needed to determine how this approach can be standardized and integrated into routine preoperative and postoperative RVF management.

## 5. Conclusions

UCAE demonstrated high sensitivity and good concordance with intraoperative findings in this cohort, suggesting its potential utility in the assessment of RVF. It may be particularly useful for detecting superficial and dynamically patent tracts. MRI remains an important complementary modality for evaluating high or complex fistulas and providing detailed soft-tissue characterization. Overall, a multimodal imaging approach combining TPUS/UCAE and MRI may offer a more comprehensive strategy for preoperative evaluation of rectovaginal fistula. Future multicenter studies are warranted to further validate these findings and explore the role of advanced imaging technologies in clinical practice.

## Figures and Tables

**Table 1:** Suggested surgical approaches based on combined ultrasound classification (institutional proposal).

Category (Anatomic Location + Morphology + Size)	Suggested Surgical Approach (Route)
Low + single tract + small	Transvaginal or transanal tract excision + primary closure
Low + branched tract + medium	Transperineal or transvaginal tract excision + Martius flap repair ± sphincteroplasty
Middle + complex tract + medium/large	Transabdominal tract excision + Martius or gracilis flap ± sphincteroplasty
High + single tract + medium	Laparoscopic or open abdominal repair ± temporary diversion
High + multiple tracts + large	Open or laparoscopic tract excision + gracilis muscle transfer + multiple tract closure ± ileostomy
Middle + single tract + small + sphincter defect	Transvaginal tract excision + sphincteroplasty
High + small + levator ani defect	Transabdominal tract excision + Martius flap ± pelvic reconstruction ± multidisciplinary (gynecology/colorectal)

Notes: Surgical recommendations are based on combined transperineal and contrast-enhanced ultrasound classifications. ± indicates procedures performed as needed depending on intraoperative findings.

**Table 2:** Baseline characteristics of patients with rectovaginal fistula (RVF).

Variable		Value
Age (years)		41.5 ± 9.7
Body mass index		27 ± 8.4
Etiology	Obstetric injury	35
	Inflammatory bowel disease	8
	Tumor-related therapy	10
	Surgical complications	9
Disease duration (months)	<20	38
	≥20	24
Treatment history	Conservative treatment	14
	Previous surgery	18
	Other treatments (e.g., biologics)	6

Values are presented as mean ± standard deviation (SD) for continuous variables and as counts for categorical variables.

**Table 3:** Diagnostic performance of imaging modalities for rectovaginal fistula (RVF).

Imaging Modality	Fistula Present *n* (%)	Fistula Absent *n* (%)	Total Cases	Detection Rate % (*n*/*N*)
Contrast-enhanced CT	13 (54.2)	11 (45.8)	24	54.2 (13/24)
Contrast-enhanced MRI	18 (69.2)	6 (30.8)	26	69.2 (18/26)
Barium enema	4 (66.7)	2 (33.3)	6	66.7 (4/6)
UCAE	60 (96.8)	2 (3.2)	62	96.8 (60/62)

Data are presented as counts with percentages in parentheses. Detection rate was calculated using intraoperative findings as the gold standard. Not all patients underwent every imaging modality, and some patients received more than one imaging examination. Therefore, the total number of cases differs among imaging modalities, and percentages were calculated within each imaging modality.

**Table 4:** Diagnostic performance of TPUS and MRI for pelvic floor injury assessment in the available study subgroup.

Modality	Structure	Sensitivity (95% CI)	Accuracy (95% CI)	AUC (95% CI)
MRI	Levator ani	0.55 [0.322–0.783]	0.654 [0.463–0.839]	0.775 [0.637–0.914]
	Anal sphincter	0.667 [0.435–0.863]	0.769 [0.589–0.894]	0.833 [0.709–0.957]
TPUS	Levator ani	0.810 [0.661–0.907]	0.871 [0.769–0.945]	0.905 [0.849–0.961]
	Anal sphincter	0.842 [0.710–0.925]	0.903 [0.811–0.957]	0.921 [0.877–0.965]

Abbreviations: MRI, magnetic resonance imaging; TPUS, transperineal pelvic floor ultrasound; AUC, area under the curve; CI, confidence interval.

**Table 5:** Distributional concordance between preoperative imaging and intraoperative findings.

Classification		CT (*n* = 24)	MRI (*n* = 26)	Barium Enema (*n* = 6)	UCAE (*n* = 62)	Intraoperative Findings
Morphology	Type I	11 (45.8%)	15 (57.7%)	6 (100.0%)	32 (51.6%)	33
	Type II	2 (8.3%)	5 (19.2%)	NA	21 (33.9%)	22
	Type III	0 (0.0%)	1 (3.8%)	NA	7 (11.3%)	7
Diameter	Small (<0.5 cm)	6 (25.0%)	10 (38.5%)	NA	49 (79.0%)	51
	Medium (0.5–2.5 cm)	3 (12.5%)	4 (15.4%)	2 (33.3%)	7 (11.3%)	7
	Large (>2.5 cm)	4 (16.7%)	4 (15.4%)	4 (66.7%)	4 (6.5%)	4
Rectal orifice location	High	4 (16.7%)	5 (19.2%)	4 (66.7%)	6 (9.7%)	7
	Middle	6 (25.0%)	4 (15.4%)	2 (33.3%)	31 (50.0%)	32
	Low	3 (12.5%)	9 (34.6%)	NA	23 (37.1%)	23

Overall comparison: χ^2^ = 23.91, df = 3, *p* < 0.001. Data are presented as counts with percentages in parentheses. NA = not applicable.

## Data Availability

The data presented in this study are available on request from the corresponding authors. The data are not publicly available due to privacy or ethical restrictions.

## Abbreviations

The following abbreviations are used in this manuscript:

AUC	Area under the curve
BMI	Body mass index
CI	Confidence interval
CT	Computed tomography
ERUS	Endorectal or endovaginal ultrasound
MRI	Magnetic resonance imaging
ROC	Receiver operating characteristic
RVF	Rectovaginal fistula
TPUS	Transperineal pelvic floor ultrasound
UCAE	Ultrasound contrast agent enema
